# Endothelial cells-derived exosomes-based hydrogel improved tendinous repair via anti-inflammatory and tissue regeneration-promoting properties

**DOI:** 10.1186/s12951-024-02607-0

**Published:** 2024-07-09

**Authors:** Yichen Dou, Hong Zhai, Haiqiu Li, Hanlin Xing, Cheng Zhu, Zhaopeng Xuan

**Affiliations:** 1 Department of Hand and Podiatric Surgery, Orthopedics Center, The First Hospital of Jilin University, Jilin University, 130031 Changchun, P.R. China; 2 Department of Laboratory Medicine, The First Hospital of Jilin University, Jilin University, 130031 Changchun, P.R. China

**Keywords:** Tendon healing, HUVECs-exos, Inflammation, TDSCs, Macrophage polarization

## Abstract

Tendon injuries are common orthopedic ailments with a challenging healing trajectory, especially in cases like the Achilles tendon afflictions. The healing trajectory of tendon injuries is often suboptimal, leading to scar formation and functional impairment due to the inherent low metabolic activity and vascularization of tendon tissue. As pressing is needed for effective interventions, efforts are made to explore biomaterials to augment tendon healing. However, tissue engineering approaches face hurdles in optimizing tissue scaffolds and nanomedical strategies. To navigate these challenges, an injectable hydrogel amalgamated with human umbilical vein endothelial cells-derived exosomes (HUVECs-Exos) was prepared and named H-Exos-gel in this study, aiming to enhance tendon repair. *In our research involving a model of Achilles tendon injuries in 60 rats, we investigated the efficacy of H-Exos-gel through histological assessments performed at 2 and 4 weeks and behavioral assessments conducted at the 4-week mark revealed its ability to enhance the Achilles tendon’s mechanical strength, regulate inflammation and facilitate tendon regeneration and functional recovery.* Mechanically, the H-Exos-gel modulated the cellular behaviors of macrophages and tendon-derived stem cells (TDSCs) by inhibiting inflammation-related pathways and promoting proliferation-related pathways. Our findings delineate that the H-Exos-gel epitomizes a viable bioactive medium for tendon healing, heralding a promising avenue for the clinical amelioration of tendon injuries.

**Supplementary Information:**

The online version contains supplementary material available at 10.1186/s12951-024-02607-0.

## Introduction

Tendons act as pivotal conduits between muscles and bones, which orchestrate movement and facilitate intricate functions [1, 2]. Regrettably, tendon afflictions are exceedingly prevalent among orthopedic ailments, with the highest occurrence rates being observed among military personnel, athletes, and the broader populace. For instance, in the United States, upwards of 800,000 individuals necessitate surgical interventions on an annual basis due to injuries pertinent to this type of detriment [3, 4]. Emerging research has underscored a rising trend in the incidence of the Achilles tendon rupture, probably attributed to the growing elderly population [5]. The recuperation of impaired tendons seldom attains full restoration or reclamation of their inherent function post-healing, which can be ascribed to the tendon’s inherent low metabolic activity, minimal oxygen uptake, sparse vasculature, diminished cellularity, and the chasm engendered by the retraction of the ruptured distal extremity [6–9].

Tendon injuries often result in significant pain and disability, thus imposing severe clinical and financial burdens on the society [10]. Despite strides in regenerative medicine, effective treatments remain lacking because the tendons have limited natural healing capacity, which can be ascribed to their poor cell density and vascularization. The advent of tissue engineering heralds promises in regenerating tendon-like tissues that display compositional, structural, and functional characteristics akin to native tendon tissues [6, 7, 11]. This regenerative discipline endeavors to restore physiological tissue functions through amalgamating cells and materials, and is augmented by suitable biochemical and physicochemical factors. Current strategies encompass biomaterials, scaffold fabrication techniques, cells, biological adjuncts, mechanical loading, and bioreactors, among which, macrophage polarization plays a notable role in tendon regeneration [12]. Nevertheless, the persistent challenges warrant future explorations to enhance the efficacy and longevity of tendon injury treatments [13].

The prevailing remedial avenues for tendon injuries leave much to be desired; for instance, autografts frequently culminate in secondary injuries, while allografts, xenografts, and ligament prostheses are associated with the risk of immunological refutation [14–16]. Succinctly, tissue transplantation achieves suboptimal performance, necessitating protracted recuperation durations for patients [7]. Some research has harnessed the potential of stem cells, dermal fibroblasts, and tenocytes to bolster tendon regeneration [6, 7, 9]. Nonetheless, obstacles including immunological refutation, cell sourcing, and ethical quandaries circumscribe the applicability of exogenous cell transplantation [17, 18]. Furthermore, this modality may precipitate ectopic ossification [19].

In recent epochs, the domain of regenerative medicine has been galvanized by the burgeoning intrigue surrounding the therapeutic potential of exosomes, particularly those derived from human umbilical vein endothelial cells (HUVECs), in the arena of tissue repair and regeneration. Exosomes, small vesicles with a diameter spectrum of 50 to 150 nm, are pivotal in cell communication, carrying microRNAs, mRNAs, and proteins that facilitate repair and regeneration across various tissues, including tendons, muscles, and nerves [20, 21]. Recent scholarly endeavors have highlighted the therapeutic potential of HUVECs-derived exosomes (HUVECs-Exos) in the realm of cutaneous wound healing and skin regeneration, where HUVECs-Exos are utilized to augment the proliferative and migratory activities of keratinocytes and fibroblasts, the two cardinal effector cells instrumental for skin regeneration. The strategic integration of HUVECs-Exos with a gelatin methacryloyl hydrogel scaffold, when applied in full-thickness cutaneous wounds, not only orchestrates the repair of wound defects but also facilitates the sustained release of exosomes, leading to the accelerated re-epithelialization, enhanced collagen maturity, and improved angiogenesis [22]. Additionally, the mechanistic underpinnings of HUVECs-Exos in oxidative stress have been explored, unveiling their influential role in ameliorating the survival of skin flaps through modulating the comportment of endothelial progenitor cells (EPCs) [23]. This evidence suggests HUVECs-Exos could revolutionize tissue repair strategies, offering new solutions for orthopedic and dermatological conditions.

The booming realm of macrophage polarization has emerged as a focus in the orchestration of tissue repair, notably through modulating inflammatory cascades and fostering stem cell proliferation [12, 24, 25]. Such immunological plasticity predominantly manifests in the dichotomy of M1 and M2 macrophages, each exerting distinct functions in the inflammatory and reparative stages of tissue healing [26]. Specifically, M1 macrophages initiate a strong inflammatory response essential for clearing pathogens and debris [27]. C*onversely, M2 macrophages promote tissue regeneration by supporting extracellular matrix remodeling, angiogenesis, and fibrosis* [28, 29]. Moreover, the nuanced interplay between macrophages and stem cells amplifies the regenerative range, with macrophages modulating stem cell behavior via diverse paracrine signaling pathways, thereby augmenting stem cell proliferation and differentiation essential for tissue repair [30–32]. This synergy highlights innovative therapeutic strategies for improving tissue repair and reducing inflammation, marking significant progress in regenerative medicine [33].

**Fig. 1:**
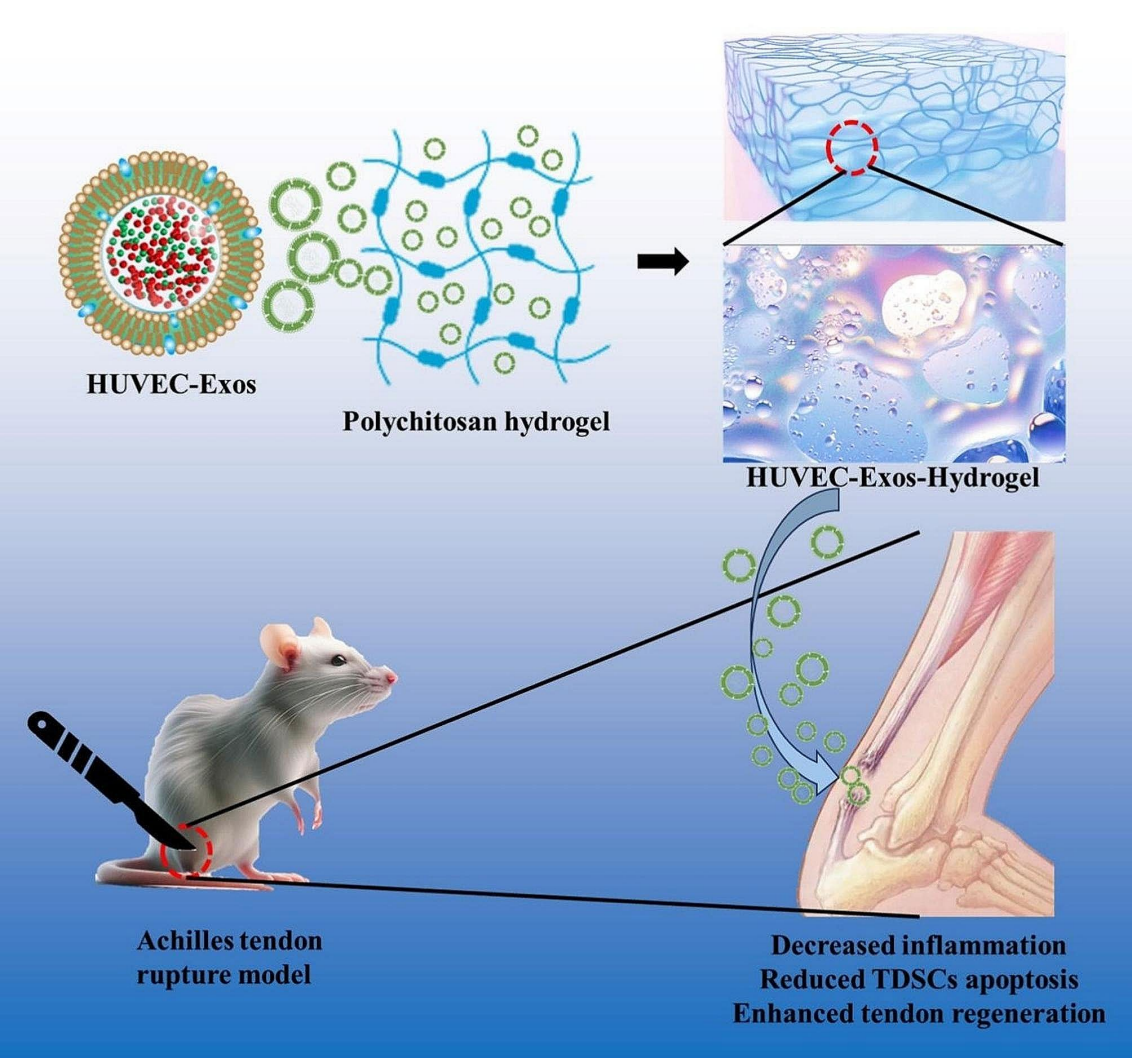
Schematic diagram of the application of HUVECs-Exos hydrogel to promote tendon regeneration from three aspects

Predicted upon these contemplations, HUVECs-Exos were prepared and applied in the remediation of tendon injuries in this study. According to the results, HUVECs-Exos were effective on amplifying mechanical fortitude, reducing the inflammatory responses, accelerating tendon regeneration, and promoting functional recuperation through both in vitro and in vivo explorations. Moreover, a rat model of Achilles tendon injury was constructed for exhaustive in vivo assessment, including histological and behavioral evaluations. Our results delineate that HUVECs-Exo epitomizes a viable bioactive medium for tendon restoration, presenting a promising avant-garde strategy for the clinical amelioration of the Achilles tendon afflictions (Fig. 1).

## Materials and methods

### Ethical statement

In this study, animal experiments were performed in adherence to the guidelines for the Care and Utilization of Laboratory Animals delineated by the National Institutes of Health Guide. Moreover, the experimental protocols were approved by the Animal Ethics Committee, The First Hospital of Jilin University (jdfy2023-0614). All efforts were carried out to minimize animal suffering.

### HUVEC identification

HUVECs were purchased from the National Collection of Authenticated Cell Cultures (Shanghai, China) and were cultured using an endothelial cell medium with 10% FBS (Sciencell, USA). Thereafter, the morphology of HUVECs was characterized under an optical microscope, where HUVECs exhibited a fusiform shape, consistent with the literature [34]. Afterwards, HUVECs were fixed and conducted immunofluorescence staining with a VE-Cadherin (VE-Cad) specific antibody. Positive staining was observed, which denoted the presence and localization of VE-Cad in HUVECs. Later, HUVECs were detached, resuspended in the staining buffer, and incubated with a fluorochrome-conjugated anti-CD31 antibody. Flow cytometry analysis was performed to evaluate the expression of CD31, a pivotal endothelial marker. The positive expression of CD31 corroborated the endothelial lineage of HUVECs. To evaluate the angiogenic potential of HUVECs, an in vitro tube formation assay was carried out. Initially, Matrigel Matrix (Corning) was thawed overnight at 4 °C. Subsequently, 150 µL of Matrigel Matrix was carefully added to each well of a 48-well plate and gently mixed on ice to prevent premature gelation of the Matrigel. The plate was then incubated at 37 °C for 1 h to allow the Matrigel Matrix to solidify. Approximately 6 × 10*4 HUVECs were seeded into each well. After an 8-hour incubation period, the formation of tube-like structures by HUVECs was observed and documented using an optical microscope (SZ61TR, Olympus, Japan).

### Exosome-related methods

Exosomes were extracted with reference to a preceding study [35]. After HUVECs reached 100% confluence, the cell-containing growth medium was replaced with an exosome-depleted medium (Vivacell, China). After continued incubation for 72 h, the supernatant was meticulously collected and placed into an ultracentrifuge (Thermo Fisher Scientific, USA) for 35-min centrifugation at 10,000 g and 4℃, and later additional 45-min centrifugation at 12,000 g, so as to eliminate cell debris. Supernatants from both steps were obtained and further centrifuged at 120,000 g and 4℃ for 120 min. The supernatant was then discarded, and the resultant precipitate was resuspended. This resuspension was subsequently transferred into centrifuge tubes containing 50 ml pre-cooled 1× PBS, and potential contaminants were filtered with a 0.22-µm filter (Millipore, USA). The tubes were re-centrifuged at 120,000 g for 120 min at 4℃, the supernatant was discarded, while the precipitate was resuspended with exo-free PBS. The procured exosomes were either preserved at -80℃ or used for subsequent experiments.

Pursuant to our preceding study [35], the Nanoparticle Tracking Analysis (NTA) of exosomes was executed. By utilizing NanoSight NS300 (Malvern, U.K.), the concentration and particle size of HUVEC-EVs were analyzed based on the principles of light scattering and Brownian motion, thereby delineating particle size characteristics of the suspended samples. Data acquisition and processing were accomplished by one operator with software version V1.08, aligning with the minimal data requisites for EV studies (MISEV) (22).

According to our prior study [35], the morphological assessment of EVs was conducted by Scanning Electron Microscopy (SEM, TESCAN, China) and Transmission Electron Microscopy (TEM, FEI, USA). For SEM, 150 ul of HUVEC-EVs suspension was cooled at -20℃ overnight, and then lyophilized in a vacuum desiccator. An appropriate amount of cryogen was transferred into the sample, and the lyophilized EVs powder was spread flat, followed by ion sputtering and visualization under SEM. In TEM, the isolated EVs samples were placed on a copper grid coated with a 0.125% Formvar chloroform solution, stained with a solution of 1% (v/v) UO2 acetate dissolved in double-distilled water, and promptly imaged and assessed with TEM.

### Preparation of the HUVECs-Exos-chitosan hydrogel

The chitosan-based hydrogel was formulated by initially dissolving 100 mg of chitosan (sourced from Aladdin, China) in a 5 ml solution of 0.6% acetic acid (provided by Sinopharm Group, China), ensuring complete dissolution through continuous stirring. The solution’s pH was then adjusted to a neutral range of 7.0-7.4 using sodium hydroxide (NaOH) from Aladdin, China, to create a biocompatible environment. The exosome suspension, prepared in phosphate-buffered saline (PBS) to achieve a concentration of 2.8E + 11 particles/mL, was subsequently integrated under sterile conditions. This mixture was combined with a 0.5 ml aliquot of 2% hydroxyethyl cellulose solution (sourced from TCL, Shanghai, China), with continuous stirring until gelation occurred, resulting in the formation of what we designate as H-Exos-gel. The NC-gel was produced using an identical protocol, except for the exclusion of the exosome component.

### Cell culture

Rat tendon-derived stem cells (TDSCs) were extracted in line with the established protocols, *and have been reported to express CD44 and CD90, while showing negativity for CD31 and CD106* [36]. Briefly, pentobarbital was injected into a quartet of male Sprague-Dawley rats aged 6–8 weeks and weighing 200–250 g for anesthesia, and then the lower extremities of these rats were thoroughly disinfected. Afterwards, the Achilles tendon was excised along with the per-tendinous connective tissue, and the residual tissue was precisely fragmented in phosphate-buffered saline (PBS), digested with 3 mg/mL collagenase type I (Gibco, USA) for 3 h, and agitated at 37 °C at a velocity of 200 rpm. The ensuing single-cell suspensions were obtained by filtering out the undigested tissue fragments with a 70-µM cell strainer (Corning, USA). After centrifugation at 1200 rpm, cells were re-suspended in the DMEM/F-12 medium supplemented with 10% fetal bovine serum (FBS) and 1% penicillin-streptomycin solution (Gibco, USA), then plated and incubated in a CO2 incubator at 37 °C overnight. The medium was changed every other day, and a trypsin-EDTA solution (Gibco, USA) was added to digest cells in passage 0 (P0) on day 7. Cells in passage 3 were used in all experiments.

The RAW264.7 macrophage cell line was acquired from the Cell Bank of the Chinese Academy of Sciences, Shanghai [12]. The cells were cultured in the high-glucose DMEM (Gibco, USA) supplemented with 10% FBS and 1% penicillin-streptomycin solution (Gibco, USA), and incubated in a humid incubator under 5% CO2 and 37℃ conditions.

### Exosomal microRNAs sequencing

Micro-RNA sequencing was completed through a commercial conduit (Personal Biotechnology Co., Ltd. Shanghai, China) [37]. In brief, total RNAs were extracted from *three separate batches* of *HUVECs-Exos* with the miRNA easy Kit (Qiagen, Hilden, Germany). Then, the quality and concentration of the extracted total RNAs were assessed by Qubit (Life Technologies, USA) Agilent 2200. After reverse-transcription-PCR amplification, micro-RNA libraries were created for sequencing, and raw reads were obtained with Illumina HiSeq 2500 platform. Thresholds of |log2 (fold change)| ≥ 1 and *p* < 0.05 were applied in the classification of differentially expressed entities.

### Bioinformatics analyses

Differentially expressed miRNAs were calculated and filtered with the threshold of q value < 0.05 and FC > 2 or FC < 0.5. The q value was calculated using the DEG algorithm in the R package for experiments with biological replicates. The targets of differentially expressed miRNAs were predicted using the software Miranda in animals, with the parameters as follows: S ≥ 150, ΔG ≤ -30 kcal/mol, and demanding strict 5′ seed pairing. To delve deeper into the underlying biological pathways, KEGG pathway enrichment analysis was conducted based on the KEGG database (http://www.genome.ad.jp/kegg) by R software [38].

### Live-dead cell staining

The effects of NC-gel and HUVECs-Exos hydrogels on cellular viability were evaluated through using a live/dead cell staining kit (Beyotime, China) [39]. To be specific, TDSCs were inoculated into 24-well plates (5 × 10^4^ cells/well) and cultured for 24 h. Thereafter, the culture medium was added with an aqueous solution of NC-gel and HUVECs-Exos hydrogels and further incubated. At 24 h after culture, the remaining medium was discarded, and the cells were washed with PBS twice. Subsequently, the PBS mixture containing calcein AM and propidium iodide was added into each well (300 µl/well), and the resultant mixture was further cultured at 37 °C for 30 min. Subsequently, live cells, which gave the vivid green fluorescence, and dead cells, which emitted the ruby red fluorescence, were observed with a cutting-edge confocal microscope (Nikon, Japan).

### Proliferation-related assays

#### CCK-8 assay

The viability of TDSCs after intervention with NC-gel and H-Exos-gel hydrogels was evaluated by employing the Cell Counting Kit-8 (CCK-8, Beyotime, China) [12], in line with specific protocols. Briefly, TDSCs were inoculated at a density of 1 × 10^3^ cells/well in the 96-well plates (Corning, USA), and then intervened with NC-gel and H-Exos-gel hydrogels for 24 h. Subsequently,10 µl CCK-8 solution was poured into each well to incubate for another 2 h. The optical density (OD, measured at a wavelength of 450 nm) of the plates was detected with a microplate reader (Bio-Rad, USA). Finally, cell viability was calculated as the ratio of the mean OD from diverse interventions to the control OD, and the product was multiplied by 100%.

#### BrdU

The proliferation of TDSCs was observed by utilizing the 5-Bromo-2’-deoxyuridine (BrdU) Adsorption Assay Kit (Cell Signaling Technology, USA), according to the manufacturer’s protocols [12]. Briefly, TDSCs were inoculated at a density of 1 × 10^6^ cells/well in the 6-well plates, and later exposed to distinctive interventions for 24 h. At 12 h after intervention, BrdU solution was added into each well. The cells were washed with PBS, and incubated with rat anti-BrdU primary antibody at ambient temperature for 1 h. Ultimately, the cell nuclei were re-stained with 4’,6-diamidino-2-phenylindole (DAPI) for 5 min. *The visual elucidation was carried out using an immunofluorescence microscope (Leica, Germany).* The proliferation of cells was determined by the formula below: *average number of BrdU + cells/number of DAPI × 100%.*

### Macrophage polarization assessment

RAW264.7 cells were seeded at a density of 1 × 10^6^ cells/well in the six-well petri dishes, nurtured within a high-glucose DMEM (Gibco, USA) milieu, supplemented with 10% fetal bovine serum (FBS, Gibco, USA) and a 1% penicillin-streptomycin solution (Gibco, USA). After incubation for 24 h, the culture medium was replaced with a hydrogel extract supplemented with 1000 ng/ml lipopolysaccharide (LPS). Following an additional 24 h of incubation, macrophage polarization was further assessed using immunofluorescence staining. The procedure was as follows: cells were initially rinsed with PBS, fixed in 4% paraformaldehyde (Servicebio, China) for 10 min, and blocked with 5% BSA (Beyotime, China) at room temperature for an hour. Subsequently, the slides were stained overnight at 4 °C with the following primary antibodies: CD86 (1:200), CD163 (1:200), iNOS (1:200), F4/80 (1:200) (Abcam, Cambridge, UK). The next day, they were incubated for an additional hour with fluorescent secondary antibodies (Abcam, Cambridge, UK), and DAPI (Abcam, Cambridge, UK) was used for counterstaining of the nuclei. Lastly, images of 3 randomly selected fields of view magnified 50 times were captured using an immunofluorescence microscope (Leica, Germany) for subsequent statistical analysis.

### Establishment of the rat model of achilles tendon injury

All the animal experiments were approved by the Institutional Animal Care and Use Committee (jdfy2023-0614) of The First Hospital of Jilin University, meanwhile were conducted according to the Guide for the Care and Use of Laboratory Animals. All efforts were carried out to minimize animal suffering. Establishment of Tendon Injury Model and Treatment in Male Sprague-Dawley rats (6 weeks of age, approximating 200 g in weight) were arbitrarily and evenly segregated into four clusters, each comprising 15 rats: (1) Control group, (2) Model group, (3) NC-gel group and (4) H-Exos-gel group. In this study, the Achilles tendon rupture healing model described in previous literature was adopted [40]. A preliminary analgesic treatment with ibuprofen solution (30 mg/kg) (Merck, Germany) was administered, followed by anesthesia induction using pentobarbital sodium (30 mg/kg) and ketamine (5 mg/kg). Subsequently, the right hind limb of each rat was shaved and disinfected with povidone-iodine. A 1.5 cm longitudinal incision at the right ankle was made to fully expose the Achilles tendon, while the left hind limb remained uninjured. Blood seeping from the neighboring wound area was fully aspirated and the area was washed with saline solution. Following blunt dissection, the Achilles tendon was entirely transected at its midpoint. The injured Achilles tendon was then sutured using the Kessler technique, and 0.15 ml of NC-gel or H-Exos-gel was injected to coat the surface of the injured tendon, as required by the experiment. After a pause of 10–20 min, the skin incision was meticulously sutured. An additional dose of ibuprofen solution (30 mg/kg) was administered for pain management 6 h post-operation. Upon regaining consciousness, the rats were grouped into cages with five rats per cage and maintained at a room temperature of 25 °C, with daily monitoring conducted. Rats were allowed to move freely in captivity. Penicillin (200000UI/kg) (Merck, Germany) was administered for three consecutive days post-surgery to avert wound infection. At 14 or 28 days, the rats were euthanized using an excess of CO_*2*_, allowing for the removal of the right Achilles tendon.

### Gait analyses

The locomotive performance of rodents was evaluated with the CatWalk XT gait analysis apparatus (CatWalk XT; Noldus, the Netherlands) [25]. In the model and the H-Exos-gel groups, surgical maneuver was confined solely to the right hind limb, whilst the left hind limb was left untouched, so as to avoid any potential posture alterations by the rats. Since these posture alterations might interfere with the identification of disparities between the two hind limbs. On the flip side, the normal cohort did not receive any surgical treatment on either hind limb. To adapt the rats to the impending task, an intensive training regimen was formulated. To be specific, the rats were instructed to ambulate from one extremity of an enclosed passageway to the other on the top of an illuminated glass panel. The focus was continuous locomotion without any extended pause for at least one week. The rat footprints were captured by adopting an internal light footprint refraction technique. Each footprint not only depicted its area but also visually illustrated the relative pressure exerted by the foot. The light intensities emitted were in direct proportion to the extent of relative pressure. High-velocity video cameras were utilized to record the footprints, which were subsequently funneled through automated analysis with the CatWalk XT 10.0 software.

### Histological analysis

At 14 and 28 days after surgical maneuver, the right Achilles tendon of each rat was excised and fixed within a 4% paraformaldehyde (PFA) solution for 24 h [41]. After fixation, the sample was dehydrated, embedded in paraffin and sliced into the 5-µm sections. These sections were later dewaxed and rehydrated with gradient solutions of xylene/alcohol and PBS. To obtain a broad-spectrum histological insight, the sections were subjected to hematoxylin and eosin (H&E) staining, Masson’s trichrome staining, and Sirius red staining. Thereafter, the H&E staining results were evaluated by two researchers who were blinded to the experimental cohorts by using a modified Stoll scale [42]. More details of the scale are available in Supplementary Table 1 of Supporting Information. The results of Masson’s trichrome staining, with the assistance of Image J, allowed for the identification of collagen tissues via the strong brightness in the grayscale. Measurements were taken from five selected areas, and then an average was calculated.

Fragments of the Achilles tendon were obtained by segmenting the Achilles tendon samples into 5-µm-thick slices, as described earlier [43]. *After deparaffinization and rehydration, samples were fixed and antigen repaired using trypsin. The extraneous water was removed, and each tissue sheet was marked with circles utilizing a highlighter.* The Achilles tendon fragments were later submerged in a solution consisting of 5% BSA and 0.5% Triton-X-100 (Servicebio, China) for 1 h at room temperature. *Following this incubation, samples were incubated overnight at 4 °C with the primary antibody: CD86 (1:200), CD163 (1:200), iNOS (1:200), F4/80 (1:200),(Abcam, Cambridge, UK).* Thereafter, the samples were washed with 1 × TBST thrice for 5 min each, both before and after another 1 h incubation with Alexa Fluor 488 anti-rat (H + L) or Alexa Fluor 594 anti-rat (H + L) secondary antibody (1:500; Abcam, UK) at ambient temperature. The results were visualized with an immunofluorescence microscope (Leica, Germany). *The fluorescence intensity in each region was calculated using Image J software.*

### Biomechanics analysis

At 14 and 28 days after surgery, the *Sprague-Dawley rats* were humanely euthanized and later the fresh Achilles tendons were excised. Both adhering tissues and adjacent muscular structures to the Achilles tendon were removed, preserving solely the untouched regenerated tendon for further analysis. The tensile strength of the regenerated Achilles tendon was gauged with a universal testing apparatus (Instron, UK). Then, one clamp was fastened at the junction of the muscle-tendon interface, while the other one was anchored at the junction of the Achilles tendon and bone, and a distance of 10 mm was maintained between the two clamps. To prevent any slippage during the test, a finely interlaced gauze was integrated as a cushioning substance. Afterwards, the sample was elongated at a pre-defined tensile rate of 15 mm/min [14]. Key metrics including peak load, tensile resilience, elastic modulus, and rigidity of each sample were obtained from the load-elongation diagram. In particular, data regarding the load-to-failure (N) ratio were collected from the diagram. Furthermore, the linear segment of the diagram was examined to acquire the elastic modulus (MPa), and its relational dynamics with strain and cross-sectional area (CSA) were elucidated.

### Biosafety evaluation

To measure the biosafety of H-Exos-gel, a nuanced experimental protocol was enacted [44, 45]. At 14 and 28 days after surgery, the Achilles tendon was dissected from each rat, besides, the heart, liver, spleen, lung, and kidney tissues were also removed. After gentle cleaning, the collected samples were fixed in a 4% PFA solution for 24 h. Thereafter, the samples were thoroughly dehydrated, embedded in paraffin, and prepared into the 5-µm-thick sections. These sections were dewaxed and rehydrated with xylene/alcohol and PBS solutions. To comprehensively assess the biosafety of the material, sections were conducted H&E staining, Masson’s trichrome staining, and immunofluorescence staining, respectively.

### Statistical analysis

Each experiment was conducted in triplicate. To ensure the objectivity and reliability of our assessments, all experiments were conducted with the assessors blinded to the identity of the groups being evaluated. This blinding procedure was rigorously maintained throughout the data collection and analysis phases. GraphPad Prism 9 was used for data analysis. The two-group comparison was analyzed using two-tailed Student’s t-test analysis, whereas a one-way analysis of variances (ANOVA) followed by a Bonferroni post-hoc test was used to compare several groups. Significance levels were set at *p < 0.05, **p < 0.01, and ***p < 0.001.

## Results

### Identification of HUVECs and HUVECs-Exos

**Fig. 2:**
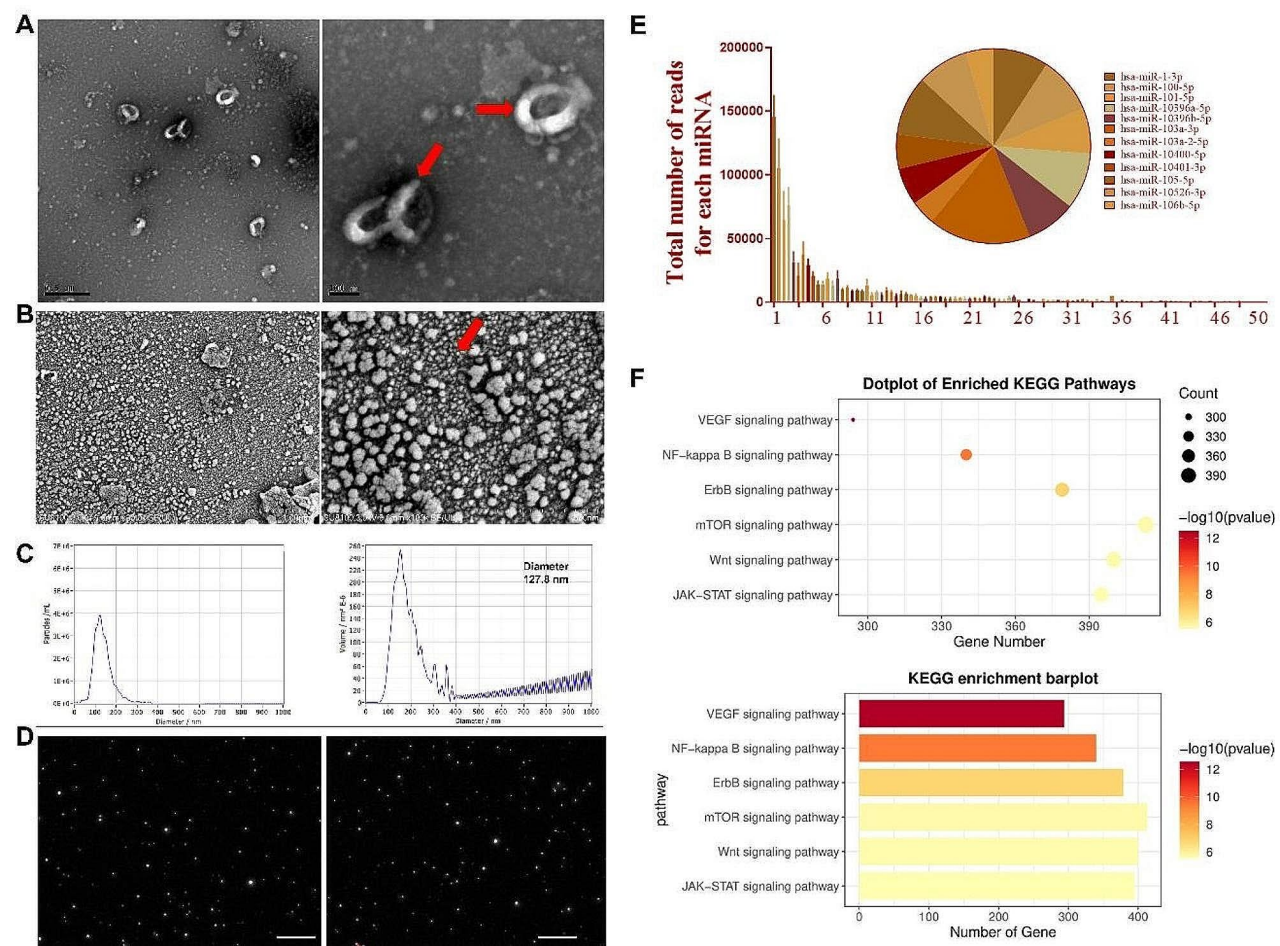
Identification of HUVECs-Exos and its cargo. (**A**) Transmission electron microscopy with scale bars of 2.5 μm and 100 nm; (**B**) scanning electron microscopy with scale bars of 5 μm and 500 nm; (**C-D**) particle-size analysis; (**E**) miRNAs sequencing of HUVECs-Exos and top 10 expressed miRNAs; (**F**) KEGG analysis of miRNAs from HUVECs-Exos

First, HUVECs were characterized to ensure the quality and specificity of the subsequently extracted exosomes. As a result, HUVECs showed a shuttle shape under light microscopy, consistent with literature reports (Supplementary Fig. 1A). In addition, the positive staining for VE-Cad/Occludin and positive flow cytometry analysis for CD31/CD105 (negative for CD45) further demonstrated the purity of HUVECs (Supplementary Fig. 1B-C). Furthermore, a conventional differential centrifugation method was employed to collect HUVECs-Exos. Under TEM and SEM observations, HUVECs-Exos presented a typical goblet or spherical shape (Fig. 2A and B). Additionally, NTA results demonstrated that the diameters ranged from 30 to 150 nm (Fig. 2C), and the percentage of molecules within this range was 94.92%, with a diameter interval is 127.8 ± 21.7 nm. Meanwhile, the original concentration of HUVECs-Exos was 1.22 × 10^10^/ml (Fig. 2D).

### Sequencing and bioinformatics analyses of HUVECs-Exos

To examine the anti-fibrotic effects of HUVECs-Exos, exosomal microRNA sequencing was carried out among the three different HUVECs-Exos groups. The integrity and uniformity of each sample were validated by using different techniques (Supplementary Figs. 2 and 3). Notably, the top ten most significantly expressed miRNAs were detected, including let-7a-5p, let-7a-3p, let-7b-5p, and let-7b-3p (Fig. 2E). Through KEGG pathway enrichment analysis, several signaling pathways were enriched, among which, the *mTOR* signaling pathway (a known pro-fibrotic signaling pathway) was the most noteworthy (Fig. 2F). These findings suggest that HUVECs-Exos are involved in the anti-fibrotic effects.

### H-Exos-gel promotes the M2 polarization of macrophages and reduces their M1 polarization in *vitro*

**Fig. 3:**
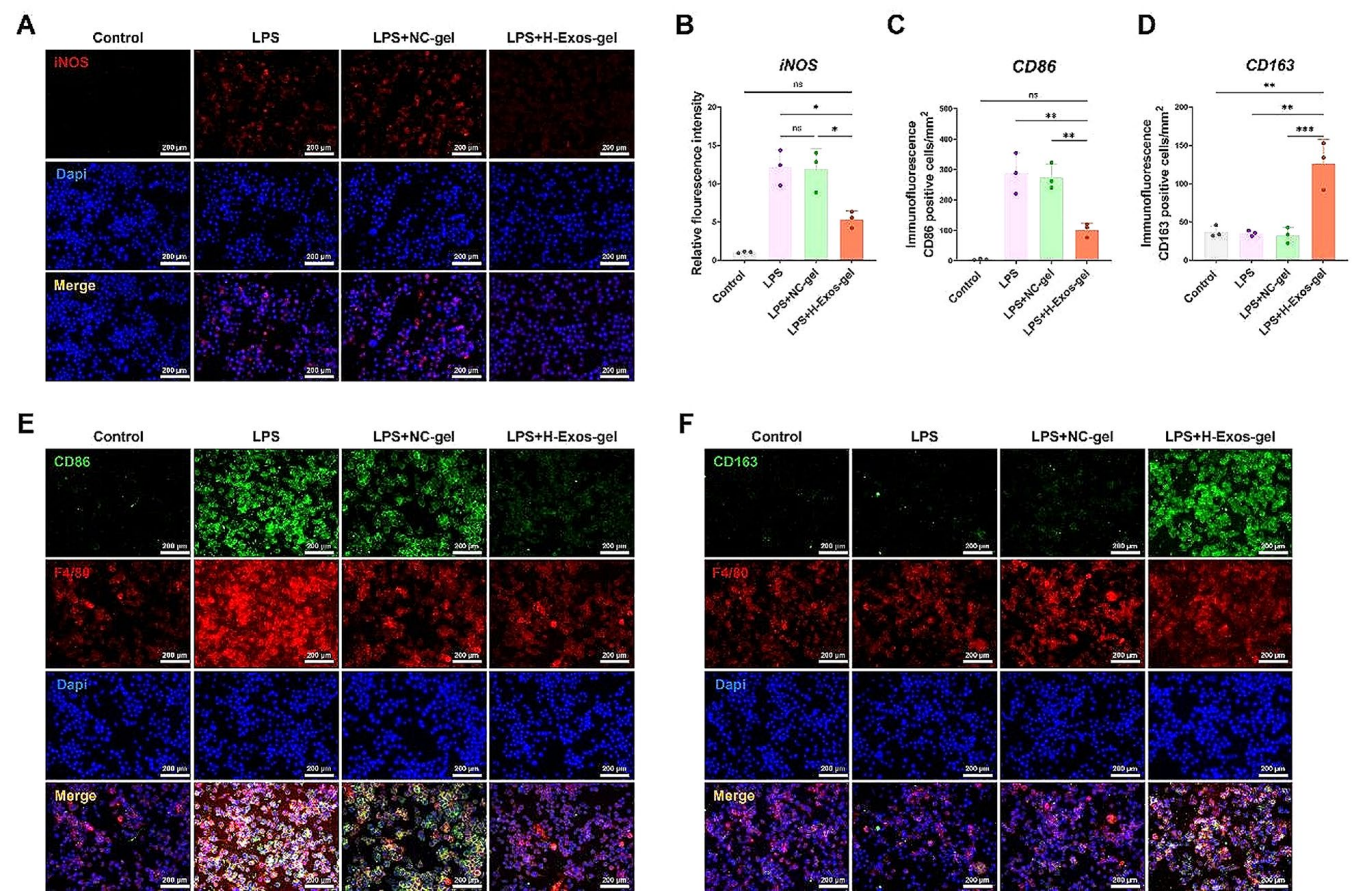
H-Exos-gel promotes the polarization of macrophages towards the M2 phenotype while inhibiting polarization towards the M1 phenotype. (**A**) Schematic diagram of different treatments for RAW264.7 and immunofluorescence analysis was utilized to assess the relative expression and spatial distribution of iNOS under different treatment regimens. Scale bar = 200 μm. (**B**) The relative fluorescence intensity of iNOS following diverse treatments (*n* = 3) was assessed. The data are depicted as the mean ± SD. The statistical significance of the disparities between the various treatments was evaluated using a one-way ANOVA with the Bonferroni post-test. Notably, statistical significance was denoted as NS: no significance, *p < 0.05. (**C-D**) Quantification of CD86 and CD163 positive cells following different treatments (*n* = 3). The data are presented as mean ± SD. Statistical significance was determined using one-way ANOVA with Bonferroni post-test. Significance levels are denoted as NS: no significance, ***p < 0.001 and **p < 0.01. (**E**) Immunofluorescence analysis was employed to ascertain the relative expression and spatial distribution of CD86 and F4/80 under various treatment conditions. Scale bar = 200 μm. (**F**) Immunofluorescence analysis was employed to ascertain the relative expression and spatial distribution of CD163 and F4/80 under various treatment conditions. Scale bar = 200 μm

The inducible nitric oxide synthase (iNOS), an enzyme predominantly found in immune cells like macrophages, plays a pivotal role in the immune responses of macrophages and the management of inflammation. In this study, immunofluorescence staining was conducted to detect the iNOS expression. Remarkably, just one day after LPS induction in *macrophages*, the LPS Control group exhibited a pronounced expression level of iNOS. Conversely, in the NC-gel group, the iNOS expression remained consistently high, without any noticeable variation. The H-Exos-gel group demonstrated a significant decrease in iNOS expression, revealing the potential of H-Exos-gel in mitigating inflammatory responses in macrophages (Fig. 3A-B). As suggested by immunofluorescence staining and relative quantification, the number/unit area/per square mm of CD86 + F4/80 + M1 macrophages markedly elevated in the LPS and NC-gel groups, while the ratio significantly decreased in the H-Exos-gel group (Fig. 3C and E). Concurrently, the above-mentioned ratio of CD163 + F4/80 + M2 macrophages in the LPS, NC-gel, and Control groups remained low, while that in the H-Exos-gel group markedly increased (Fig. 3D and F).

### H-Exos-gel promotes the proliferation of TDSCs and reduces their death in *vitro*

**Fig. 4:**
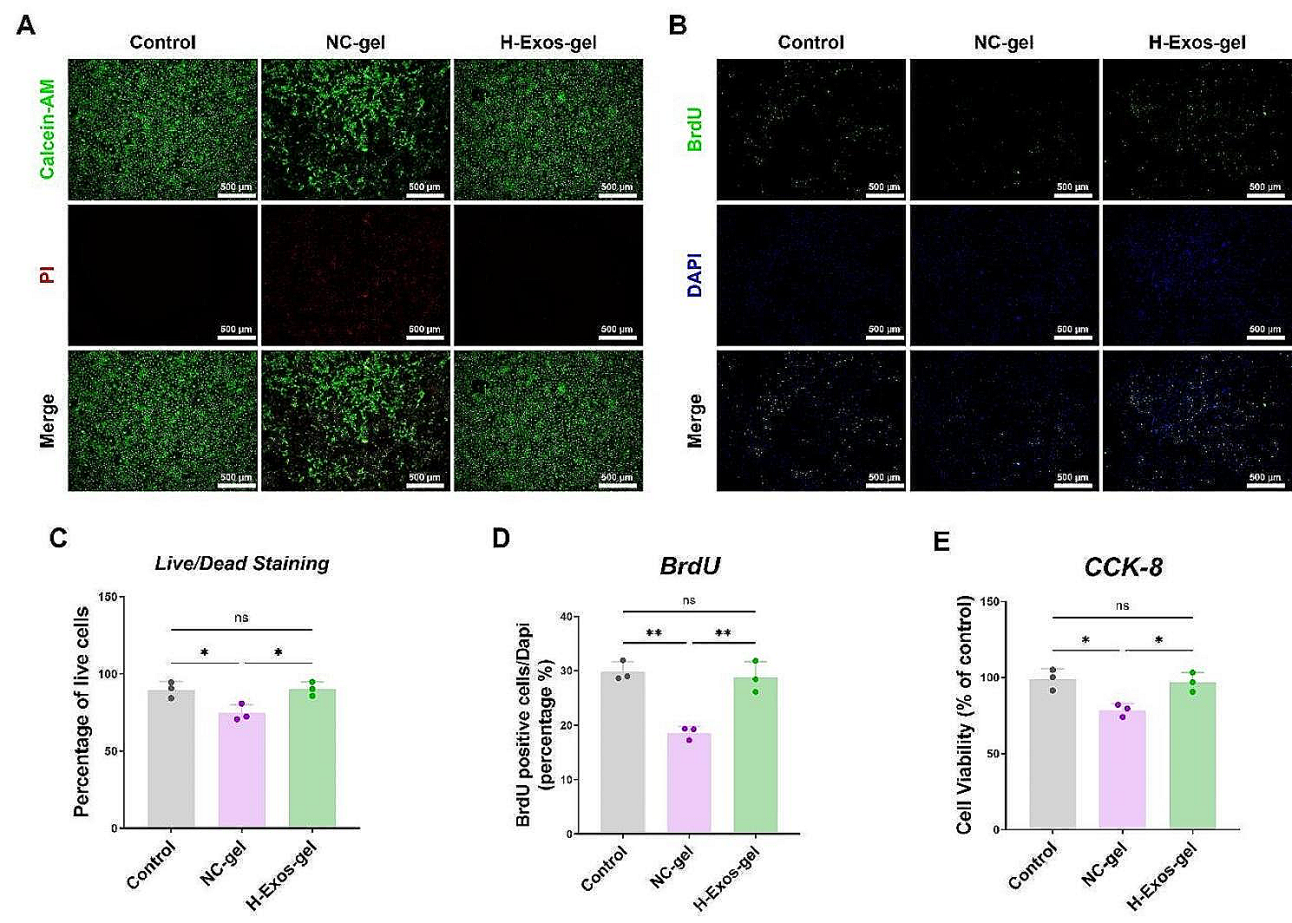
Cellular-level biocompatibility evaluation of H-Exos-gel. (**A**) Live/Dead assay evaluation of TDSCs (green, live; red, dead) proliferation and cytotoxicity cultured with H-Exos-gel at 7 days. Scale bar = 500 μm. (**B**) The cell proliferation capacity of TDSCs was assessed through a BrdU doping assay after 24 h of distinct treatments. Green signal = BrdU. Scale bar = 500 μm. (**C**) Survival of TDSCs (*n* = 3). Data are presented as mean ± SD. The statistical significance of the differences between various treatments was determined by one-way ANOVA with Bonferroni post-test. NS: no significance and *p < 0.05. (**D**) Quantification of BrdU assay data (*n* = 3). Data are presented as mean ± SD. NS: no significance and **p < 0.01. (**E**) Cell proliferation ability of TDSCs was further determined using CCK-8 assay. (*n* = 3) Data are presented as mean ± SD. NS: no significance and *p < 0.05

Following a 7-day culture of TDSCs (tendon-derived stem cells) on H-Exos-gel and NC-gel hydrogels, the survival rate of TDSCs was evaluated using a live/dead cell staining kit. On day 7, TDSCs cultured on H-Exos-gel retained their polygonal form, which was in striking contrast to the sparse well-spread cells observed on the NC-gel scaffold. Besides, the cell mortality rate on the NC-gel scaffold surged, plummeting the live cell ratio to around 74%. Regardless of a marginal increase in cell mortality rate on the H-Exos-gel, the live cell ratio maintained above 90%, accompanied by enhanced cell spreading (Fig. 4A and C).

The CCK8 assay revealed the decreased OD values of both the NC-gel and H-Exos-gel groups compared with that of the Control group. Typically, a more obvious decrease was observed in the NC-gel group, whilst a slight downtrend was detected in the H-Exos-gel group, and the difference was statistically significant between the two groups (Fig. 4E). BrdU assay indicated a continual augment in the proliferating cells of both the NC-gel and H-Exos-gel groups as time unfolded. However, a more obvious increase in proliferating cells was seen in the H-Exos-gel group compared with the NC-gel group, closely consistent with the results of the Control group (Fig. 4B and D).

### H-Exos-gel promotes the Achilles tendon repair in *vivo*

The grouping of animals forin *vivo* experiments, along with the number of animals per group and the time points, is presented in Supplementary Fig. 4.

**Figure 5:**
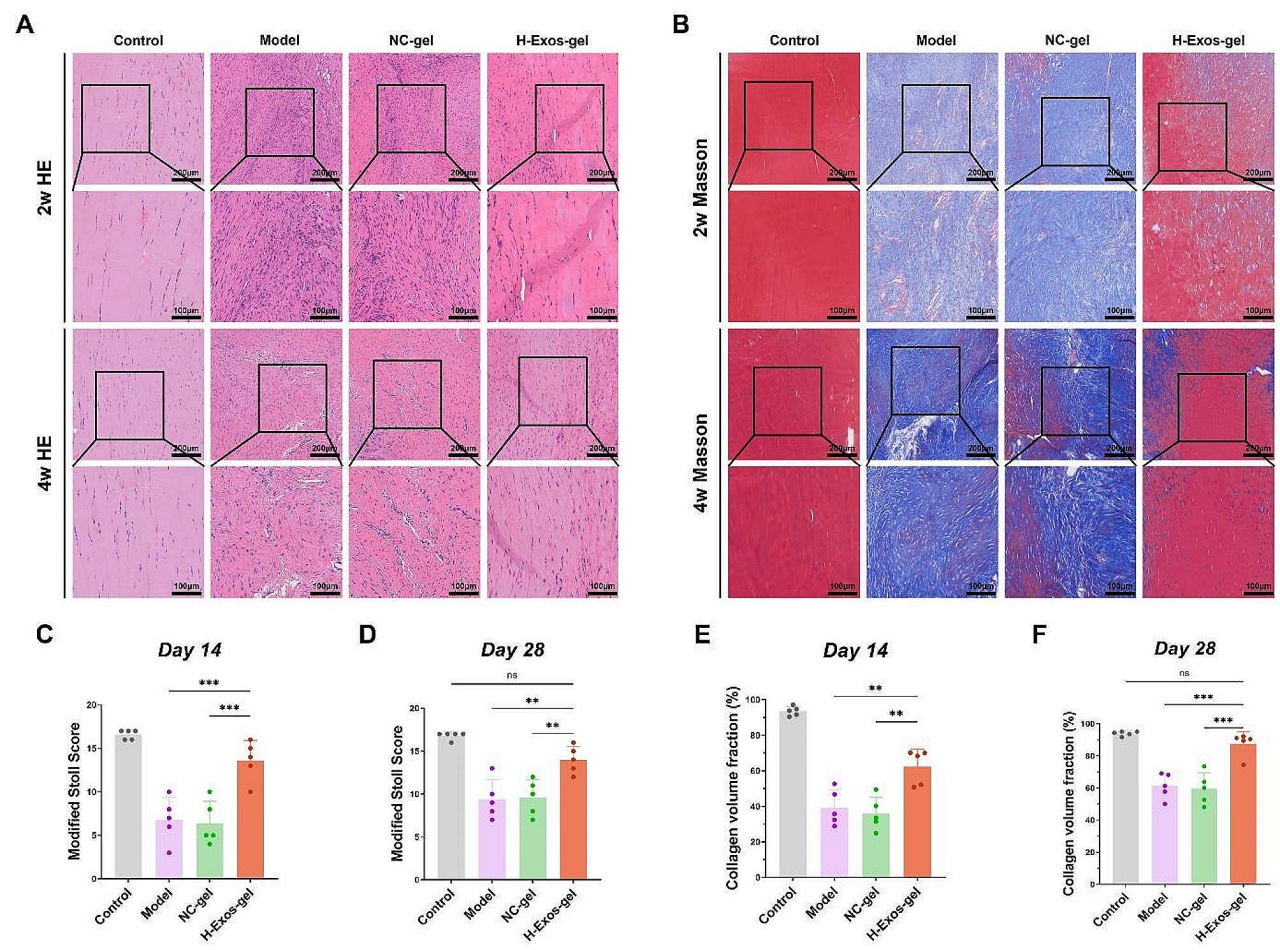
The therapeutic efficacy of H-Exos-gel for tendon injury in rats. (**A**) Representative histological images of Achilles tendons stained with HE staining from each experimental group at 2 weeks and 4 weeks. Scale bar = 200 μm–100 μm. (**B**) Representative histological images of Achilles tendons stained with Masson staining from each experimental group at 2 weeks and 4 weeks. Scale bar = 200 μm–100 μm. (**C-D**) A quantitative assessment of HE staining was conducted utilizing the refined Stoll score (*n* = 5). Data are presented as mean ± SD. The statistical significance of treatment differences was determined using one-way ANOVA with Bonferroni post hoc test. ***p < 0.001 and **p < 0.01. (**E-F**) Quantitative assessment of the collagen volume fraction through meticulous analysis of Masson-stained samples (*n* = 5). Data are presented as mean ± SD. The statistical significance of treatment differences was determined using one-way ANOVA with Bonferroni post hoc test. ***p < 0.001 and **p < 0.01

Two and 4 weeks after the surgical procedure, general observations of the tendon and H&E staining demonstrated that the majority of the material from the H-Exos-gel group was decomposed (Fig. 5A). Two and 4 weeks after surgery, the tendons that regenerated in the H-Exos-gel group displayed more aligned cellular structures and longitudinally structured fibers than those in the model group and the NC-gel group (Fig. 5A). Additionally, the enhanced presence of vasculature was evident in the model group and the NC-gel group 2 weeks postoperatively, suggesting the suboptimal tendon healing (Fig. 5A). The regenerated tendons were histologically analyzed using the revised Stoll Score. As a result, the score of the H-Exos-gel group, based on this metric, was 13.6, overshadowing those of 6.8 in the model group and 6.4 in the NC-gel group (*p* < 0.0001) 2 weeks following intervention. In week 4, the score was 14 in the H-Exos-gel group, while those were 9.4 in the model group and 9.6 in the NC-gel group (*p* < 0.001) (Fig. 5C-D). Upon Masson’s trichrome staining, the H-Exos-gel group exhibited the denser accumulation of freshly generated collagen and had better-structured fiber alignments than the control group (Fig. 5B). Two weeks after the intervention, the collagen volume fraction of the H-Exos-gel group (62.3%) was notably superior to those of 39.3% in the model group and 35.9% in the NC-gel group (*p* < 0.001). By the end of week 4, the H-Exos-gel group maintained the leading collagen volume fraction of 87.5%, in comparison with those of 61.3% in the model group and 59.6% in the NC-gel group (*p* < 0.0001) (Fig. 5E-F).

### H-Exos-gel promotes macrophage polarization towards the M2 phenotype in vivo

**Fig. 6:**
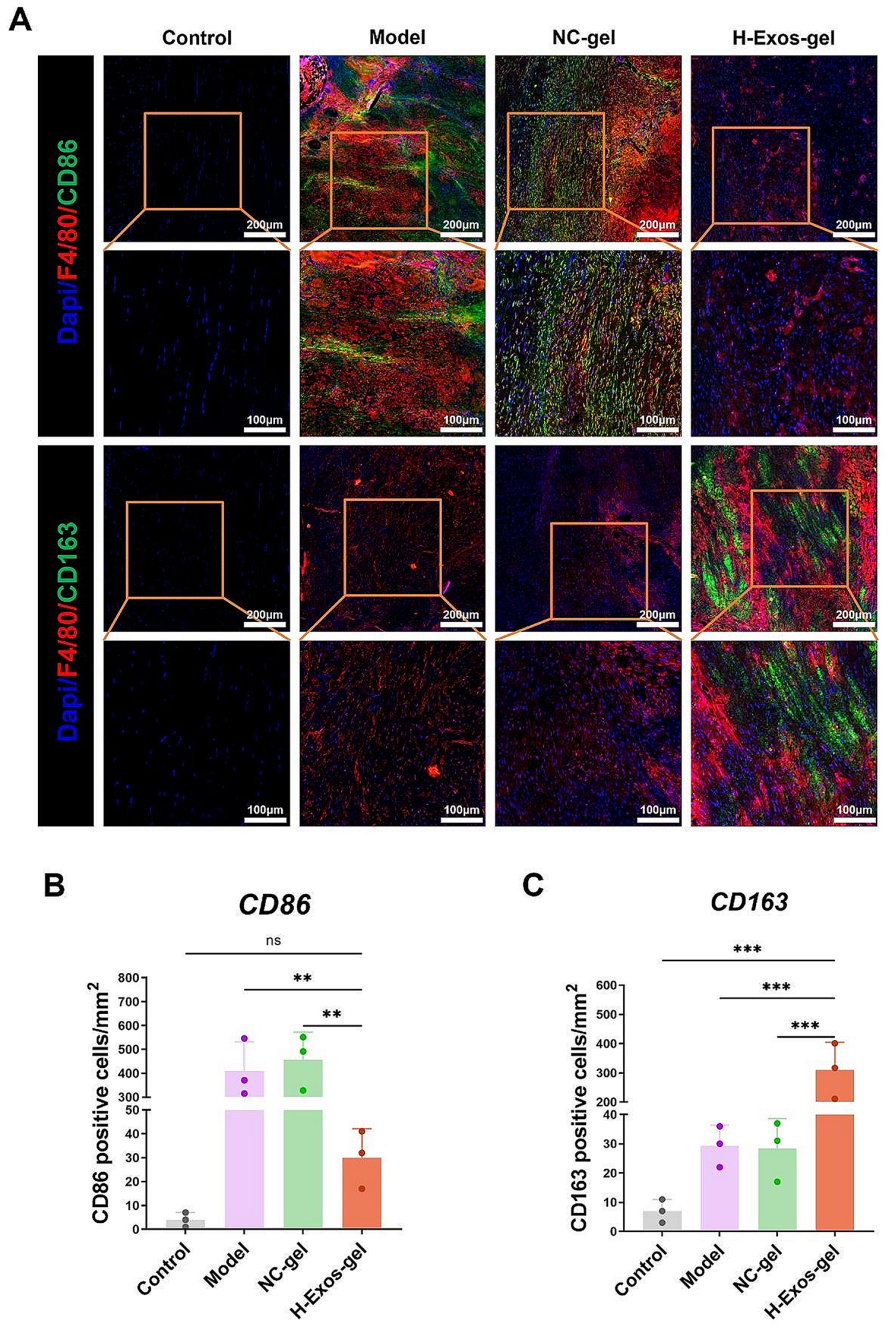
Immunofluorescence staining of rat Achilles tendons. (**A**) Immunofluorescence was used to detect the relative number and distribution of CD86, CD163, and F4/80 in different groups. Scale bar = 200 μm–100 μm. (**B-C**) Quantification of CD86 and CD163 positive cells following different treatments (*n* = 3). The data are presented as mean ± SD. Statistical significance was determined using one-way ANOVA with Bonferroni post-test. Significance levels are denoted as NS: no significance, ***p < 0.001 and **p < 0.01

Two weeks after surgery, there were more M2 macrophages (CD163+) in the H-Exos-gel group, while the number of M1 macrophages (CD86+) was noticeably lower. These findings conformed to those obtained from cellular tests, suggesting that the H-Exos-gel facilitated the control of inflammation, hence boosting tissue regeneration (Fig. 6A-C).

### H-Exos-gel promotes the recovery of the Achilles tendon function in *vivo*

**Fig. 7:**
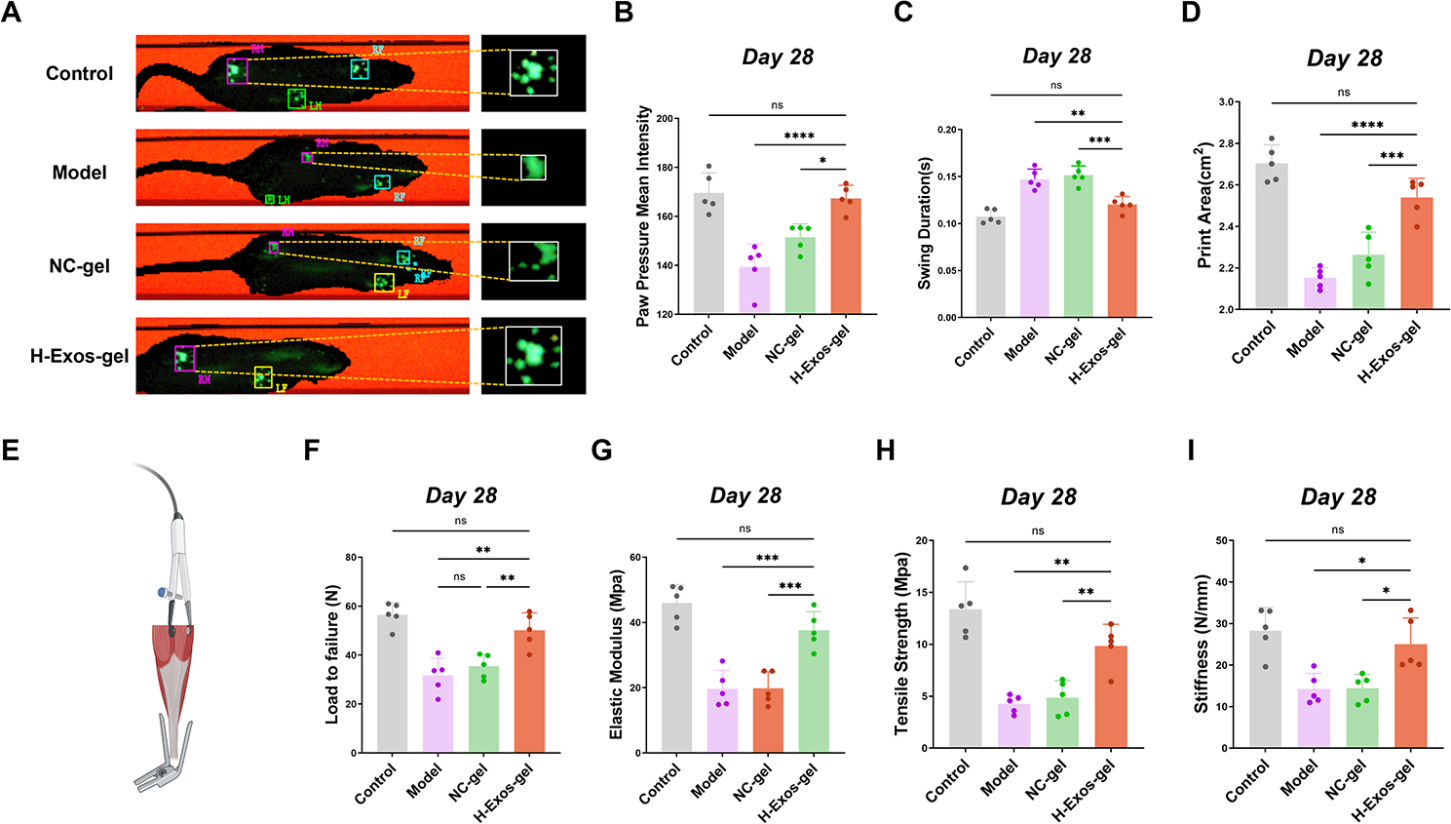
Gait testing and Biomechanical test of tendon. (**A**) Maximum contact areas of the right hind limbs at two and four weeks following the surgical intervention. (**B-D**) Quantitative analysis of three gait parameters of the hindlimb, including paw pressure mean intensity, paw print area, and swing time (*n* = 5). Data are presented as mean ± SD. The statistical significance of the differences between various treatments was determined by one-way ANOVA with Bonferroni post-test. NS: no significance, *p < 0.05.**p < 0.01, ***p < 0.001 and ****p < 0.0001. (**E**) Schematic diagram of biomechanical test. (**F-I**) Quantitative analysis of four biomechanical test parameters, including load-to-failure values, stiffness, tensile strength, and elastic modulus (*n* = 5). Data are presented as mean ± SD. The statistical significance of the differences between various treatments was determined by one-way ANOVA with Bonferroni post-test. NS: no significance, *p < 0.05.**p < 0.01 and ***p < 0.001

To assess the functional recovery of the Achilles tendon, the walking patterns of rats from the model group, NC-gel group, H-Exos-gel group, and the control group were examined 28 days after surgery (Fig. 7A-D). A closer resemblance to the standard group was deemed as a positive indicator for functional recovery. The CatWalk XT gait analysis apparatus (CatWalk XT; Noldus, the Netherlands) was utilized to monitor the locomotive performance of each rat. Besides, essential parameters including peak paw touch surface area, mean intensity of paw pressure, and duration of swing phase were used as indicators for the functional recovery of the Achilles tendon in rats. In particular, the peak touch area of 2.54 cm^2^ in the H-Exos-gel group exceeded those of the model group (2.15 cm^2^, *p* < 0.0001) and the NC-gel group (2.26 cm^2^, *p* < 0.001) (Fig. 7A and D). The mean pressure exerted by the paw of the H-Exos-gel group was 166.77, surpassing those of 139.42 of the model group (*p* < 0.0001) and 151.53 of the NC-gel group (*p* < 0.05) (Fig. 7B). Furthermore, the duration of swing phase in the H-Exos-gel group was 0.12 s, which was shorter than those of 0.15 s in the model group (*p* < 0.01) and 0.15 s in the NC-gel group (*p* < 0.001) (Fig. 7C). Collectively, these results indicate the role of H-Exos-gel in enhancing functional recovery of the damaged tendons.

During the biomechanical evaluation conducted 28 days after surgery, the load-to-failure ratio of the tendon in the H-Exos-gel group notably reached 50.14 N, which significantly eclipsed those recorded in the model group (31.66 N, *p* < 0.01) and the NC-gel group (35.3 N, *p* < 0.01), as depicted in Fig. 7F. Further, the tendon stiffness in the H-Exos-gel group was recorded at 25.03 N/mm, dramatically surpassing those of the model group (14.26 N/mm, *p* < 0.05) and the NC-gel group (14.44 N/mm, *p* < 0.05) (Fig. 7I). In terms of tensile strength, the tendon in the H-Exos-gel group demonstrated a substantial strength of 9.85 MPa, considerably outperforming those of 4.26 MPa in the model group (*p* < 0.01) and 4.86 MPa in the NC-gel group (*p* < 0.01) (Fig. 7H). Moreover, the elastic modulus in the tendon of the H-Exos-gel group was 37.63 MPa, greatly exceeding those of the model group (19.73 MPa, *p* < 0.001) and the NC-gel group (19.86 MPa, *p* < 0.001), as observed from Fig. 7G. In essence, by the 28th day after surgery, all biomechanical parameters of the H-Exos-gel group closely mirrored those obtained in the control group. Simultaneously, the regenerated tendons in the H-Exos-gel group demonstrated higher tensile strength, elastic modulus, and maximum load-bearing capacity compared to those in the Model and NC-gel groups, thus lowering the probability of tendon re-rupture. The augmented hardness of the tendon in the H-Exos-gel group could be beneficial, which facilitated the more effective force transmission from muscles to bones, thereby enhancing the running efficacy [46]. To summarize, the H-Exos-gel significantly bolsters the mechanical characteristics of the regenerated tendon.

### H-Exos-gel shows good biocompatibility in *vivo*

Histological evaluations highlighted the pronounced structural resilience of the H-Exos-gel when introduced into a live organism. Four weeks after the implantation of the H-Exos-gel, vital organs like the heart, liver, spleen, lungs, and kidneys demonstrated no notable alterations compared with the control group (Supplementary Fig. 5A). In parallel, Masson’s trichrome staining of heart, liver, spleen, lung, and kidney tissues revealed no collagen accumulation or peripheral lymphocyte clusters (Supplementary Fig. 5B). Likewise, the F4/80 immunofluorescence staining did not detect any invasion of inflammatory cells in these organs (Supplementary Fig. 5C). These histological findings authenticate the biocompatibility and safety of the H-Exos-gel in vivo.

## Discussion

The intricacy of tendon repair post-injury is underscored by the dual-edged sword of inflammation and scar formation. While inflammation is a quintessential response to injury, excessive inflammatory processes can exacerbate scar formation, thereby impairing the biomechanical integrity and functional recovery of the tendon. Scar tissue, through a natural repair mechanism, lacks the mechanical property of the uninjured tendon, predisposing it to recurrent injuries and chronic dysfunction. Besides, the inherent bradytrophic and hypovascular nature of tendon tissue further augments the challenges in tendon healing, as a result, innovative therapeutic strategies are needed to modulate inflammation and mitigate scar formation. In this regard, it is crucial to unveil the molecular mechanism underlying tendon healing and develop strategies to control maladaptive inflammation and scarring, so as to advance tendon repair mechanisms and propel the frontiers of regenerative medicine in the fields of orthopedics and sports medicine [17, 47–49].

HUVEC-derived exosomes, also referred to as HUVECs-Exos, significantly enhance the proliferation of cells and the production of collagen type 1, and are promising in managing photo-aged skin conditions [50]. Additionally, they also exhibit anti-inflammatory properties by promoting HUVEC proliferation and reducing inflammation and apoptosis, particularly through the overexpression of miR-221-3p that attenuates the HUVEC injury-induced inflammatory responses. Moreover, HUVECs-Exos possess anti-fibrotic effects by reducing the activation of hepatic stellate cells, thereby mitigating hepatic fibrosis. These findings highlight the therapeutic potential of HUVECs-Exos in managing a spectrum of pathological conditions including skin aging, hepatic fibrosis, and inflammatory disorders, and suggest that it is important to further explore the molecular and cellular mechanisms governing their actions [50–53].

Macrophage polarization, delineated into M1 (pro-inflammatory) and M2 (anti-inflammatory and tissue repair) phenotypes, plays a crucial role in inflammation and fibrosis. M1 macrophages often propel the inflammatory and fibrotic processes by releasing pro-fibrotic mediators such as transforming growth factor-beta1, directly activating fibroblasts and modulating extracellular matrix turnover [26, 35, 54, 55]. Conversely, M2 macrophages are implicated in the anti-inflammatory responses and tissue repair, demonstrating their potential in therapeutic strategies for fibrotic diseases [12, 56, 57]. Exosomes, the potent modulators of macrophage polarization, can be applied in innovative therapeutic approaches. They may harbor molecular cargoes capable of swaying macrophage polarization, thereby affecting the trajectory of inflammatory and fibrotic disorders [58–60]. The intricate interplay between macrophage polarization, exosomes, and the consequent inflammatory and fibrotic responses has elucidated a compelling avenue for the novel immunomodulatory interventions [24, 61].

The signaling pathways like NF-κB, WNT, mTOR, JAK-STAT, ErbB, and VEGF are significant players in cell inflammation and proliferation [62–64]. For example, NFκB is central for the activation of pro-inflammatory genes, while crosstalk between Wnt/β-Catenin and NF-κB can modulate inflammatory responses [65, 66]. The JAK/STAT pathway is crucial for vital cellular processes, and STAT1 expression is up-regulated in HUVECs-Exos, suggesting their regulatory role in cellular communication [67, 68]. Further, the VEGF pathway, with VEGFR-2 being a key mediator, and Wnt3a can regulate endothelial cell proliferation and migration, emphasizing their significance in angiogenesis and inflammatory conditions [69, 70]. HUVECs-Exos, especially those under the oxygen-glucose deprivation conditions, exhibit neuroprotective effects against ischemia-induced injuries, demonstrating their therapeutic potential [71]. Therefore, it is significant to understand the interplay between these pathways and HUVECs-Exos, so as to unravel the novel molecular mechanisms and therapeutic strategies for managing inflammatory and proliferative disorders.

While our investigation has highlighted the therapeutic potential of H-Exos-gel in tendon repair, it is crucial to acknowledge certain limitations of our study. First, the use of a rat model of Achilles tendon injury, despite its merits for initial discovery, was somewhat different from the clinical reality, as a result, our results should be validated in larger animal models to ensure the translational fidelity. Second, only preliminary mechanistic exploration was provided here, focusing predominantly on phenotypic changes without any in-depth mechanistic interrogation. Third, comparative studies with exosomes from other sources, such as bone marrow or adipose tissue-derived mesenchymal stem cells are lacking, which limits our understanding of the unique benefits or possible synergies of different exosomes. Consequently, it is essential to address these gaps to advance the application of exosome-based therapies in tendon healing.

In considering the long-term healing effects of H-Exos-gel, it is imperative to extrapolate from the observed short-term benefits at 2 and 4 weeks post-injury. Given the hydrogel’s anti-inflammatory and tissue regeneration-promoting properties, we theorize that early application post-injury could set a foundation for sustained healing by mitigating excessive fibrosis and encouraging proper collagen alignment. Future longitudinal studies are necessary to validate these hypotheses and determine the optimal timing for H-Exos-gel application to maximize long-term tendon function and minimize degenerative changes. In addition, the majority of human Achilles tendon ruptures, as well as other tendon injuries, often occur against a background of underlying tendinopathy [72]. Tendinopathy is characterized by collagen disarray, increased non-collagenous matrix, and neovascularization, which cumulatively weaken the tendon structure [10]. Given the regenerative and anti-inflammatory effects of H-Exos-gel observed in our rat model, there is a potential for its application in human tendons with underlying tendinopathy. By promoting proper collagen organization and mitigating inflammatory responses, H-Exos-gel may not only aid in healing post-rupture but could also offer therapeutic benefits in the pre-rupture stage of tendinopathy by strengthening tendon structure and function. However, translating these benefits to human patients requires careful consideration of the differences in tendon pathology and healing between humans and rats, underscoring the need for clinical trials.

## Conclusion

In this study, H-Exos-gel is characterized by its excellent biocompatibility and bioactive properties. At the cellular level, H-Exos-gel promotes the proliferation of TDSCs, inhibits their death, suppresses the polarization of M1 macrophages, and enhances that of M2 macrophages. In a rat model of Achilles tendon injury, H-Exos-gel improves the mechanical properties, reduces inflammatory response, and facilitates tissue regeneration and functional recovery. Our study demonstrates that H-Exos-gel significantly improves tendinous repair by modulating the inflammatory response and promoting tissue regeneration at crucial phases of healing, specifically at 2- and 4-weeks post-injury. These findings underscore the therapeutic potential of H-Exos-gel for tendon injuries. All these findings suggest that H-Exos-gel may serve as a promising therapeutic material for tendon injuries with potential clinical applications.

To translate our findings into clinical applications, several hurdles must be addressed, including scalability of production, regulatory approvals, assessments of immunogenicity and safety, and cost-effectiveness. Future studies should focus on these aspects to pave the way for clinical trials and eventual therapeutic use of H-Exos-gel in tendon repair.

### Electronic supplementary material

Below is the link to the electronic supplementary material.

Supplementary Material 1

## Data Availability

Data will be made available on request.
