# Supplementary material for: Endothelial cells-derived exosomes-based hydrogel improved tendinous repair via anti-inflammatory and tissue regeneration-promoting properties
Source: J Nanobiotechnology. 2024 Jul 9;22:401. doi: 10.1186/s12951-024-02607-0 (PMC11232200; doi:10.1186/s12951-024-02607-0)
Supplement: Supplementary file 1 — Supplementary Material 1 [file 12951_2024_2607_MOESM1_ESM.docx]

Supplemental Files:

Endothelial cells-derived Exosomes-based Hydrogel Improved Tendinous Repair via anti-inflammatory and Tissue Regeneration-promoting Properties

Yichen Dou^a^, Hong Zhai^b^, Haiqiu Li^a^, Hanlin Xing^a^, Cheng Zhu^a^, and Zhaopeng Xuan^a#^

1. Department of Hand and Podiatric Surgery, Orthopedics Center, The First Hospital of Jilin University, Jilin University, Changchun 130031,P.R.China
2. Department of Laboratory Medicine, The First Hospital of Jilin University, Jilin University, Changchun 130031,P.R.China


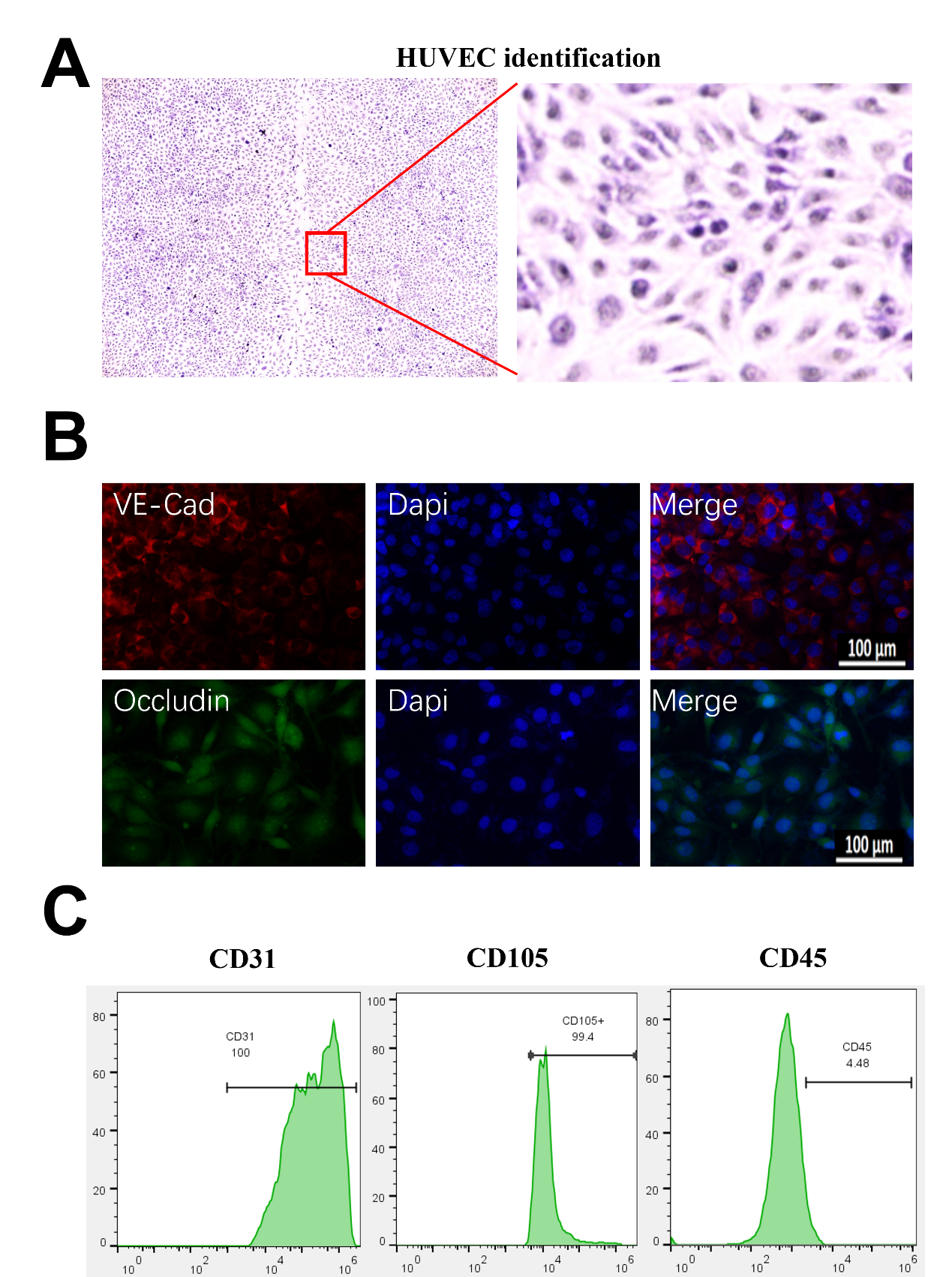


Supplementary Figure 1. Characterization and identification of HUVECs. (A) Tube formation assay for detecting the tube-forming ability of HUVECs. (B) The immunofluorescence staining indicated positive expression of VE-Cad and Occludin. Scale bar =100 μm. (C) Flow cytometry indicated positive expression of CD31, but negative expression of CD105 and CD45 in HUVECs.


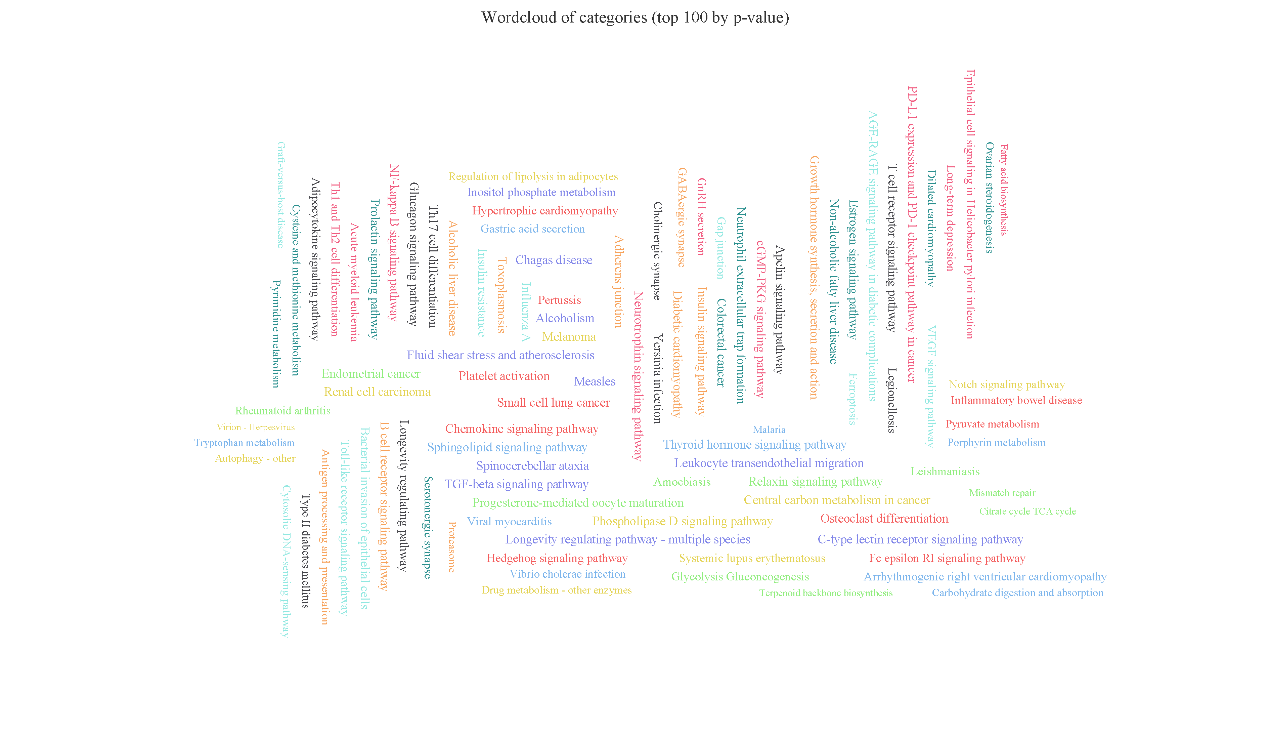


Supplementary Figure 2. Word cloud of categories or KEGG analysis of exosome miRNA cargos.


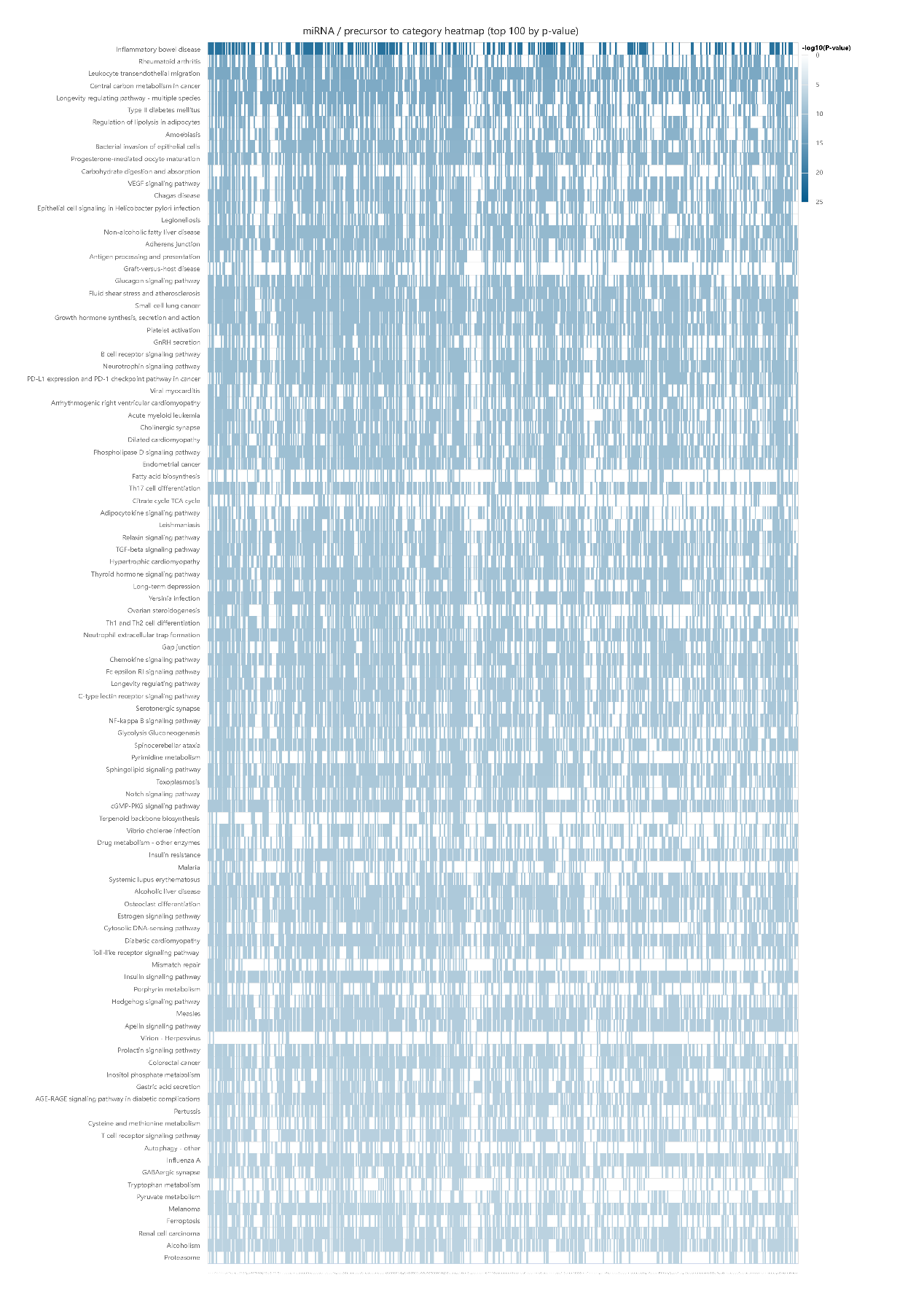


Supplementary Figure 3. Heatmap of miRNA precursor for KEGG analysis of exosome cargos.


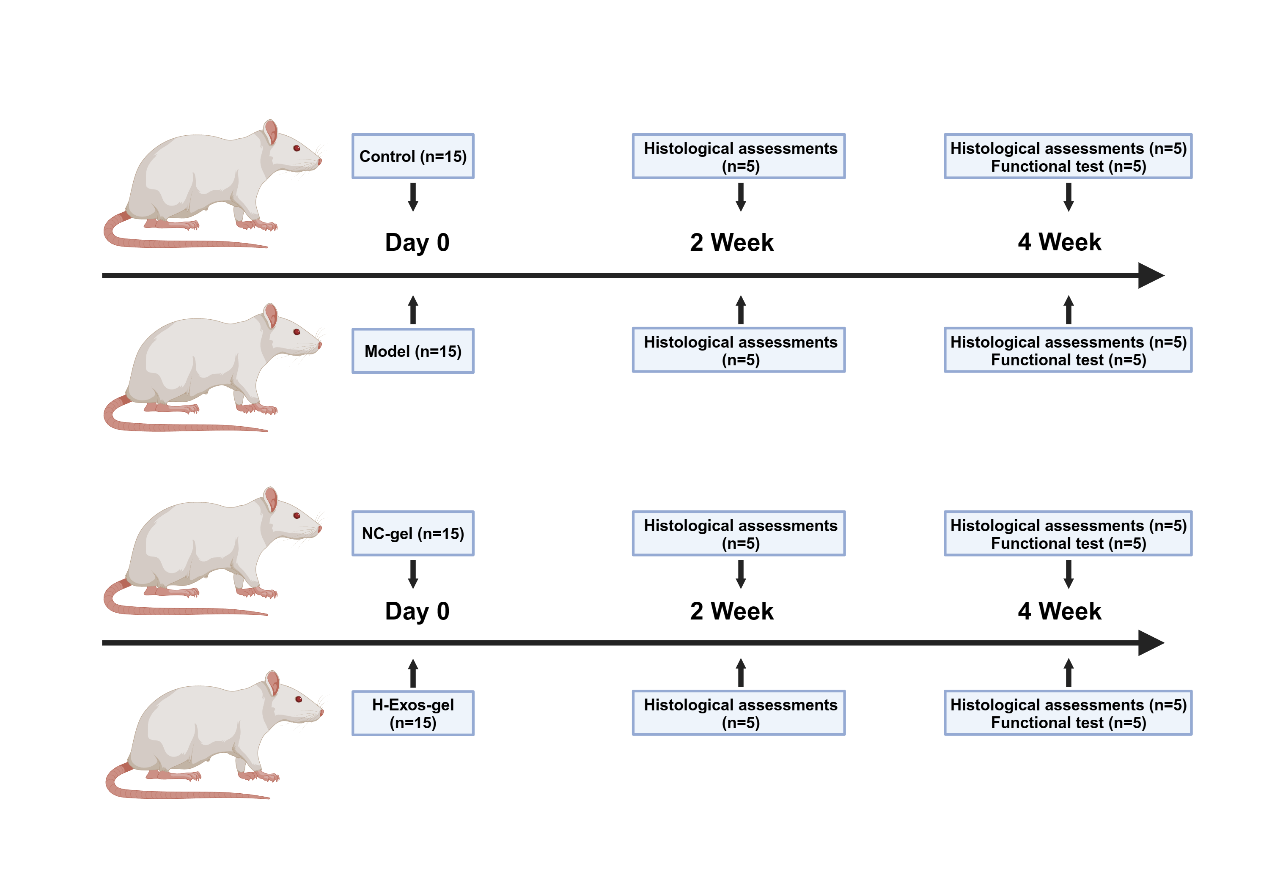


Supplementary Figure 4. Experimental flow chart in vivo including number of animals, treatment and timepoints.
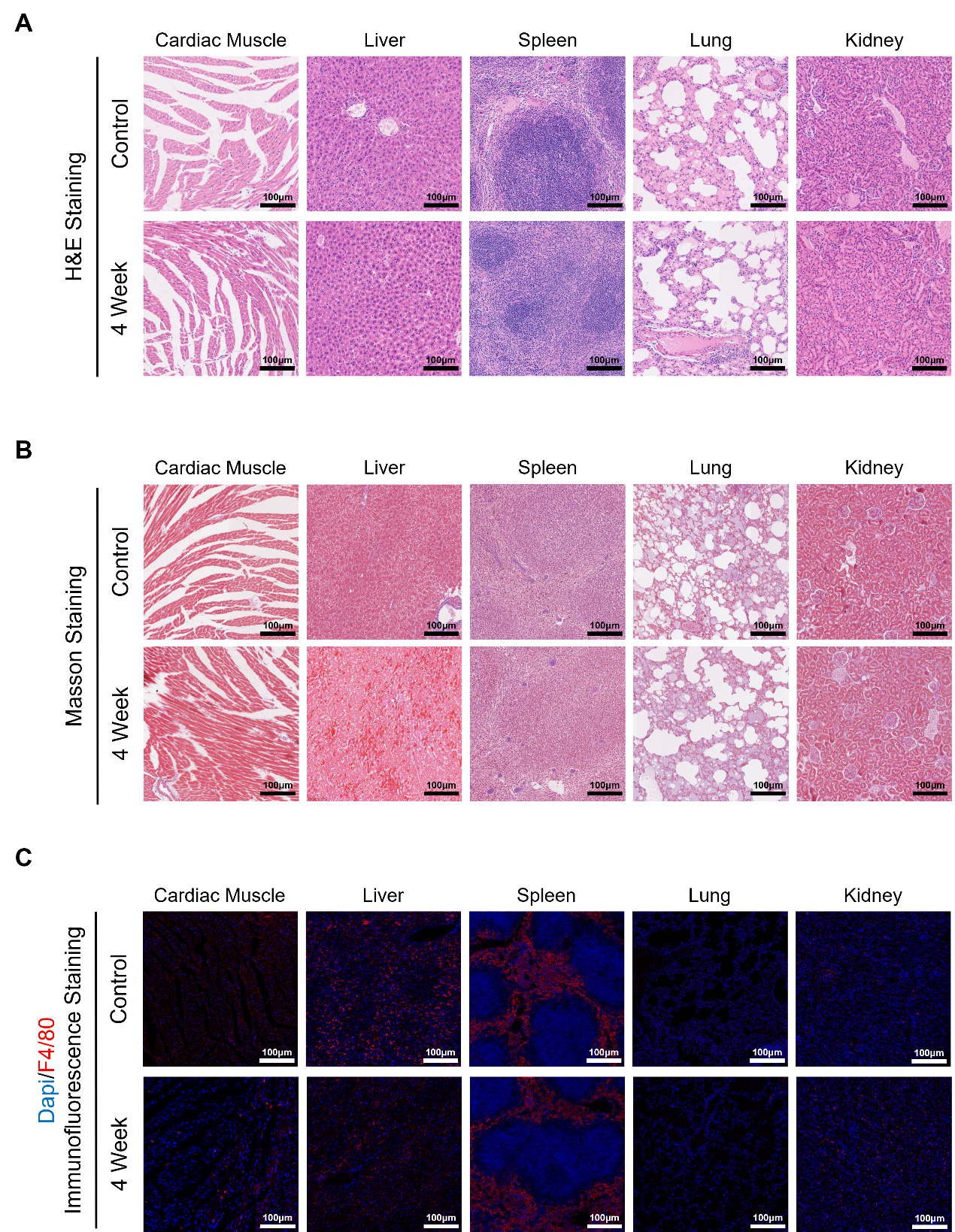


Supplementary Figure 5. Four-Week Post-Injection Analysis of H-Exos-gel Biotoxicity in vivo. (A) H&E staining of cardiac, hepatic, splenic, pulmonary, and renal tissues. Scale bar = 100 µm. (B) Masson staining of cardiac, hepatic, splenic, pulmonary, and renal tissues. Scale bar = 100 µm. (C) Immunofluorescence assay for the relative expression and spatial distribution of F4/80 within the cardiac, hepatic, splenic, pulmonary, and renal tissues. Scale bar = 100 µm.

| Evaluated parameters | Points |
| --- | --- |
| *Extracellular matrix (ECM) organization of the whole tendon* |  |
| Wavy, compact and parallel arranged collagen fibers | 2 |
| In part compact, in part loose or not orderly | 1 |
| Loosely composed, not orderly (“granulation” tissue) | 0 |
| *Cellularity/cell-matrix-ratio* |  |
| Physiological | 2 |
| Locally increased cell density | 1 |
| Increased cell density or decreased ECM content | 0 |
| *Cell alignment* |  |
| Uniaxial | 2 |
| Areas of irregularly arranged cells (10-50%) | 1 |
| More than 50% of cells with no uniaxial alignment | 0 |
| *Cell distribution* |  |
| Homogeneous, physiological | 1 |
| Focal areas of elevated cell density (cell clustering) | 0 |
| *Cell nucleus morphology* |  |
| Predominantly elongated, heterochromatic cell nuclei (tenocytes) | 2 |
| 10-30% of the cells possess large, oval, euchromatic or polymorph | 1 |
| heterochromatic nuclei |  |
| Predominantly larger, oval, euchromatic or polymorph, heterochromatic | 0 |
| nuclei |  |
| *Organization of repair tissue of the tendon callus* |  |
| Homogeneous (whole tissue with similar composition) | 2 |
| Locally heterogeneous tissue composition | 1 |
| Whole tissue composition completely changed | 0 |
| *Transition from defect to normal tissue* |  |
| Scaffold integrated, no gaps at the margin visible | 2 |
| Recognizable transition | 1 |
| Abrupt transition, splitting/gaps detectable, callus tissue | 0 |
| *Configuration of callus* |  |
| Normal, only in the defect area, locally confined | 1 |
| Strong, change of whole tendon, thickened | 0 |
| *Degenerative changes/tissue metaplasia* |  |
| Non existing | 3 |
| Moderate formation of oedema | 2 |
| Intense oedema with inclusion of fat, cell and/or fibers destruction, fibrin |  |
| deposition, gaps | 1 |
| Assembly of cartilage or bone (Safranin Orange staining) | 0 |
| *Vascularization in the defect area* |  |
| Hypo-vascularized, like surrounding tendon (small capillaries) | 1 |
| Hyper-vascularized (increased numbers of small or larger capillaries) | 0 |
| *Inflammation* |  |
| No inflammatory cell infiltrates | 1 |
| Infiltrating inflammatory cell types (neutrophils, macrophages, foreign- |  |
| body/giant cell) | 0 |

Table S1. Modified Stoll score Histological scoring system
